# Tcbf: a novel user-friendly tool for pan-3D genome analysis of topologically associating domain in eukaryotic organisms

**DOI:** 10.1093/bioinformatics/btad576

**Published:** 2023-09-19

**Authors:** Xin He, Xianhui Huang, Yuexuan Long, Zhenping Liu, Xing Chang, Xianlong Zhang, Maojun Wang

**Affiliations:** National Key Laboratory of Crop Genetic Improvement, Hubei Hongshan Laboratory, Huazhong Agricultural University, Wuhan, 430070, China; National Key Laboratory of Crop Genetic Improvement, Hubei Hongshan Laboratory, Huazhong Agricultural University, Wuhan, 430070, China; National Key Laboratory of Crop Genetic Improvement, Hubei Hongshan Laboratory, Huazhong Agricultural University, Wuhan, 430070, China; National Key Laboratory of Crop Genetic Improvement, Hubei Hongshan Laboratory, Huazhong Agricultural University, Wuhan, 430070, China; National Key Laboratory of Crop Genetic Improvement, Hubei Hongshan Laboratory, Huazhong Agricultural University, Wuhan, 430070, China; National Key Laboratory of Crop Genetic Improvement, Hubei Hongshan Laboratory, Huazhong Agricultural University, Wuhan, 430070, China; National Key Laboratory of Crop Genetic Improvement, Hubei Hongshan Laboratory, Huazhong Agricultural University, Wuhan, 430070, China

## Abstract

**Summary:**

TAD boundaries are essential for organizing the chromatin spatial structure and regulating gene expression in eukaryotes. However, for large-scale pan-3D genome research, identifying conserved and specific TAD boundaries across different species or individuals is computationally challenging. Here, we present Tcbf, a rapid and powerful Python/R tool that integrates gene synteny blocks and homologous sequences to automatically detect conserved and specific TAD boundaries among multiple species, which can efficiently analyze huge genome datasets, greatly reduce the computational burden and enable pan-3D genome research.

**Availability and implementation:**

Tcbf is implemented by Python/R and is available at https://github.com/TcbfGroup/Tcbf under the MIT license.

## 1 Introduction

With the development of Hi-C technology, researchers can now explore the three-dimensional (3D) structure of chromatin. The TAD boundaries are essential for the localization and stability of TADs, and play a key role in determining the spatial organization of the chromatin within the nucleus and regulating gene expression. The comparison of TAD boundaries between different species can provide insights into the evolution of genome organization and gene regulation (Lupiáñez *et al.* 2015, Zhang *et al.*, 2019, Pei *et al.* 2022). With the advent of the pan-genomics era, large numbers of individual reference-level genomes and Hi-C data are released, which opens up exciting new possibilities for pan-3D genomic study that requires both the comparisons of genome sequence and higher-order genome structure. When investigating the relationship of TAD boundaries between different species or individuals, the construction, interpretation, and visualization steps are complex, and no user-friendly tools are currently available for pan-3D genome analysis. One approach leverages the liftOver tool to transform the TAD boundary coordinates in one genome to those in another to analyze the conservation of the TAD boundary (Kuhn *et al.* 2013, Luo *et al.* 2021, Wang *et al.* 2022). However, generating the liftOver-dependent “chain file” is computationally expensive because of the use of lastz software to perform whole genome alignments (Harris 2007), and liftOver is designed for accurate mapping of single-base or gene coordinates, which limits the identification of large conserved TAD boundaries from complex genome regions.

Here, we developed Tcbf (Topologically associating domain Conservative Boundary Finder), a rapid and powerful Python/R tool for identifying conserved TAD boundaries. Tcbf integrates gene syntenic blocks and homologous sequences to anchor conserved boundaries, making it capable of detecting conserved TAD boundaries automatically from different species. Especially, it can identify conserved TAD boundaries from translocations and duplications. Tcbf is a command-line tool available for free at GitHub (https://github.com/TcbfGroup/Tcbf). All dependencies can be easily installed using package management tools such as PyPI (Python Package Index) and Bioconductor.

## 2 Implementation

**Figure 1.**
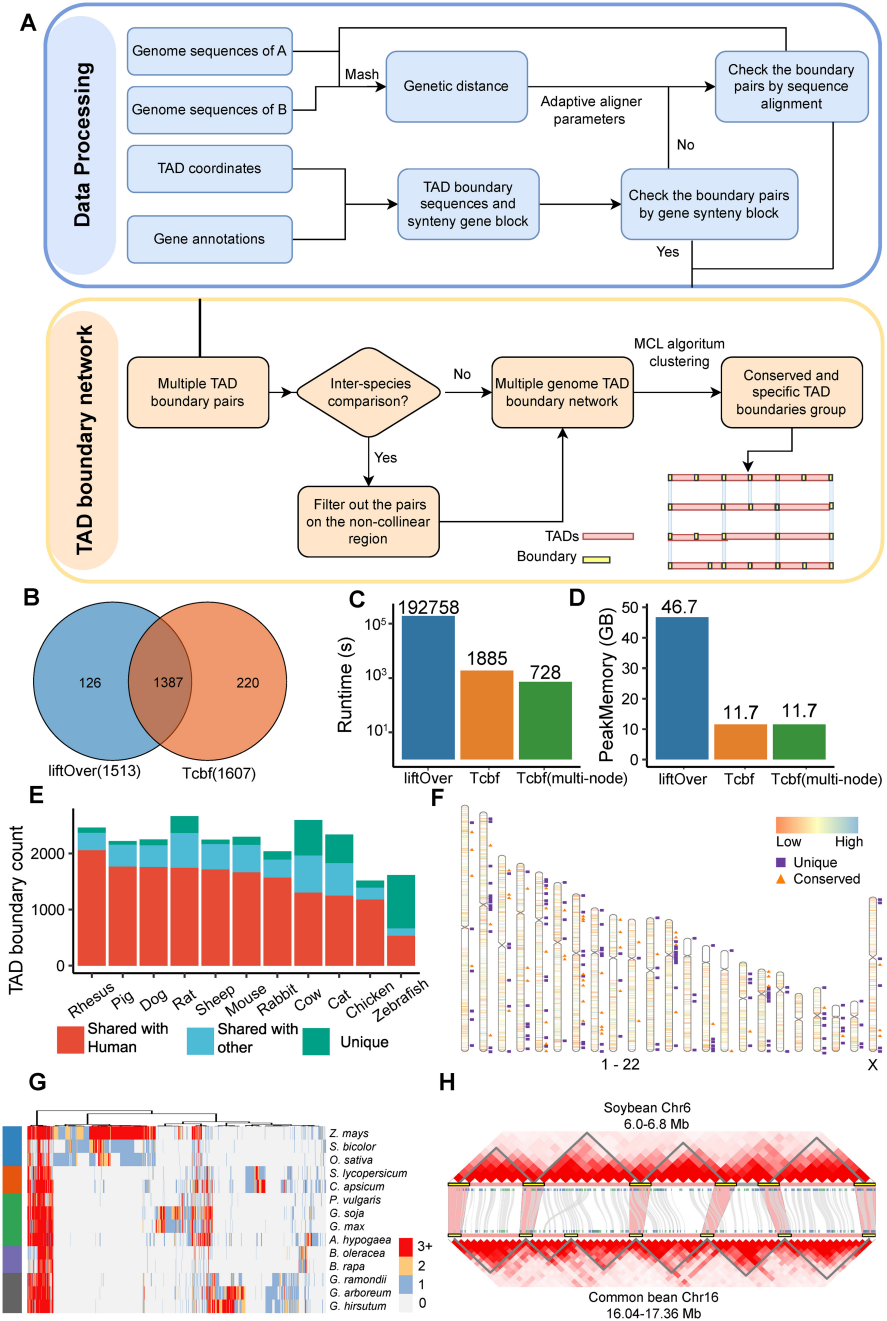
Workflow, performance profiling, and application of Tcbf. (A) Schematic model of the whole pipeline of Tcbf for inferring conserved TAD boundaries. (B and C) The performance comparison of Tcbf and liftOver “Chain” file generation. (D) Venn plot showing the difference of conserved TAD boundaries identified by Tcbf and liftOver. (E) The proportion of conserved and specific TAD boundaries in each genome. (F) The distribution position of human conserved and specific TAD boundaries. (G) Heatmap showing phylogenomic profiles of conserved TAD boundaries in 14 plants. The code to create the figure is available on an online document. (H) Representative Hi-C maps of synteny block on soybean and common bean. The shadow region connecting the TAD boundaries represents the conserved relationship of TAD boundaries.

Tcbf includes three modules: (i) dataset preprocessing, (ii) TAD boundary pair construction, and (iii) boundary graph construction, clustering, and visualization (Fig. 1A). First, the input files for Tcbf require genome sequences in FASTA format and corresponding TAD coordinates and gene annotation in GFF format. The input data are automatically preprocessed by the “first-extract-boundary” function, including cleaning up chromosome names, adding unique species identifiers, and extracting protein sequences. For species with variable TAD sizes, users can define an appropriate boundary range. Tcbf will merge and remove redundant TAD boundary coordinates and extract the genome sequence for the next process.

Second, Tcbf uses the genome of each species as the target and other genomes as the query and runs the MCScan algorithm with JCVI package to obtain syntenic gene blocks (Tang *et al.* 2008, 2017). For two TAD boundaries in two species, if orthologous gene pairs exist near the TAD boundaries, they are considered as conserved boundaries. For the TAD boundaries that do not have syntenic genes, the conservation relationships are obtained by aligning the sequences of TAD boundaries to the target genome with the minimap2 aligner (Li 2018). Since alignment results in different species can be affected by the aligner parameters, Tcbf uses the MASH distance to adjust the alignment parameters (Ondov *et al.* 2016). For example, when aligning genomes within the same species, Tcbf uses the stricter “asm5” parameter; while when aligning genomes from distantly related species, it uses the more relaxed “asm20” parameter (Supplementary File S1). After obtaining sequence alignments, Tcbf only retains alignments with length longer than 2000 bp to reduce spurious or low confidence alignments in default. If the TAD boundaries have homologous sequences, they are also considered as conserved boundaries. Finally, the identified conserved TAD boundaries from both the syntenic gene block and sequence approaches are integrated as TAD boundary pairs between the query and target genomes.

Third, the boundary pairs between multiple species are represented as a boundary graph, where each node corresponds to a TAD boundary, and the edges signify the conserved relationships between them. Edge weights are calculated as the sum of homologous gene lengths or homologous sequence length in two TAD boundary pairs. The graph is then subject to clustering using the Markov Cluster (MCL) algorithm (Dongen 2008). This analysis aims to group conserved TAD boundaries that exhibit the similar patterns of conservation across different species, which can reveal highly conserved boundary clusters across species and taxon-specific clusters. To improve the interpretability of the results, we provide a simple R script to display the results (online documents). The clusters can be visualized as a heatmap, where the color intensity represents the strength of conservation within and between clusters. This visualization method enables easy identification of conserved TAD boundary groups and facilitates the investigation of their evolutionary relationships.

## 3 Results

To evaluate the performance of Tcbf, we applied it to Hi-C data in human, mouse, and macaque from a previous study (Luo *et al.* 2021), which used liftOver to convert TAD boundary coordinates. The results from Tcbf showed that 1607 human TAD boundaries were conserved among the three species (Supplementary Table S1), slightly higher than the number (1513) reported in that study. It is found that 1387 of all boundaries were overlapped, demonstrating the consistency of the two methods (Fig. 1B). We found that the conserved boundaries reported only by Tcbf were related to genome duplication, and the usage of different aligners had an impact on the result (Supplementary File S2). The previous study did not identify conserved boundaries in duplicated genome regions because these regions were filtered out in the “chain” file generation process. We next measured the runtimes and memory usage of Tcbf and the liftOver chain file generation process (Fig. 1C and D). The average time for a complete Tcbf run was 1885 s when using 12 Intel Xeon Gold 6150 CPU cores, which is much <192 758 s (∼53.5 h) for a liftOver chain file generated with the same computing resource. In addition, since Tcbf has a scalable split task mode, if Tcbf is run on a computer cluster with 6 computation nodes, the run time is reduced to 728 s. These data show that Tcbf represents an accurate and rapid method for investigating changes in TAD boundaries between different species, especially for comparisons in complex genomic regions.

To illustrate the conserved TAD boundaries among different species, we collected TAD data from 12 vertebrate and 14 Viridiplantae species to infer 3D genome differences (Li *et al.* 2022) (Supplementary Tables S2 and S3). In vertebrates, we identified 68 conserved and 141 human-specific TAD boundary clusters in 12 species (Supplementary Table S4). The number of conserved and unique TAD boundaries was consistent with the phylogenetic distance (Fig. 1E). The human-specific TAD boundaries tended to be located in centromeric regions, especially on chromosomes 2, 8, and 12 (Fig. 1F). We identified 54 conserved TAD boundary clusters and 995 species-specific TAD boundaries in 14 Viridiplantae (Supplementary Table S5). Most of these TAD boundaries are conserved at the family level (Fig. 1G). For example, 94.6% (1176) of rice TAD boundaries were shared with maize, which is higher than the ratio of conserved genes (48.4%), suggesting that TAD boundaries might play a key role in species divergence within plant families. We also show an example of TADs in two Fabaceae plants, *Glycine max* (soybean) and *Phaseolus vulgaris* (common bean) (Fig. 1H), to demonstrate the efficiency of Tcbf. A gained TAD boundary in the common bean is located in 16.40–16.44 Mb of chromosome 16, which contains a species-specific gene Phvul.009G105850, providing an insight into the possible function of this gene at the genome topological level.

## 4 Discussion

Overall, we developed a user-friendly novel tool for pan-3D genome analysis, which is designed to be fast and accurate for identifying conserved and specific TAD boundaries among multiple species. The integration of a protein synteny block and minimap2 genome alignment to identify conserved and specific TAD boundaries is an efficient and accurate approach that reduces computing resource. The application of Tcbf will advance the study of the evolution and function of pan-3D genome organization in eukaryotes.

## Supplementary Material

btad576_Supplementary_Data

## Data Availability

The data underlying this article are available in the article and in its online supplementary data.
