# Supplementary material for: Tcbf: a novel user-friendly tool for pan-3D genome analysis of topologically associating domain in eukaryotic organisms
Source: Bioinformatics. 2023 Sep 19;39(9):btad576. doi: 10.1093/bioinformatics/btad576 (PMC10539074; doi:10.1093/bioinformatics/btad576)
Supplement: btad576_Supplementary_Data [file btad576_supplementary_data.zip › Supplementary_File_2.pdf]

The example of conserved TAD boundaries from translocations and duplications

```
human_boundary_83
human_boundary_84        mouse_boundary_1591
human_boundary_85        mouse_boundary_1590
human_boundary_86
human_boundary_87        mouse_boundary_1589
human_boundary_88        mouse_boundary_1588
human_boundary_89        mouse_boundary_1587
human_boundary_90        mouse_boundary_1586
human_boundary_91        mouse_boundary_1848
human_boundary_92        mouse_boundary_1849
human_boundary_93        mouse_boundary_1850
human_boundary_94        mouse_boundary_1850
human_boundary_95
human_boundary_96
human_boundary_97
human_boundary_98        mouse_boundary_1564
human_boundary_99        mouse_boundary_1563
human_boundary_100       mouse_boundary_1562
```

hg19 chr1 87.3 -95.9 Mb

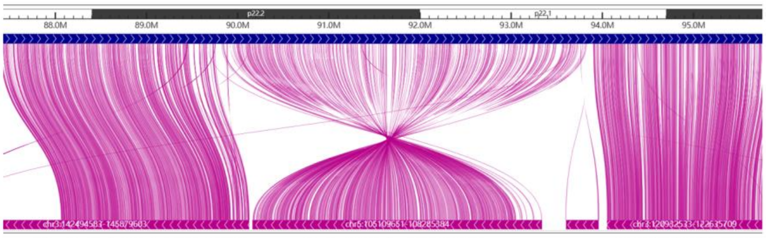

mm10 chr3 142.4-145.8 Mb   chr5 105.1-108.2 Mb   chr3 120.9-122.6 Mb

For the conserved TAD boundaries from translocation, users can check the TAD cluster by ordering the TAD boundaries of a species, and the Tcbf provides this result file. If some TAD boundaries are not in the order of the boundaries, these can be considered from the translocation. We found that mouse_boundary_1848 (chr5 105.36-105.40 Mb) was conserved with human_boundary_91 (chr1 89.92-89.96Mb), but there was no collinearity with the surrounding TAD boundaries. The genome alignment from the online website confirms the result (https://comparativegateway.wustl.edu/start/).

The example of conserved TAD boundaries from genome duplications

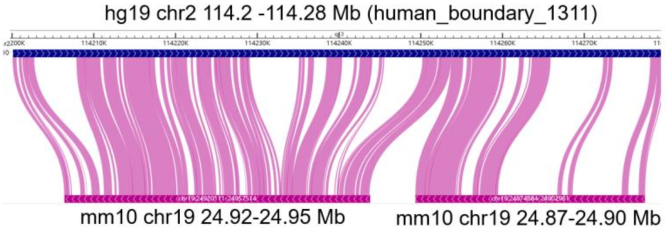

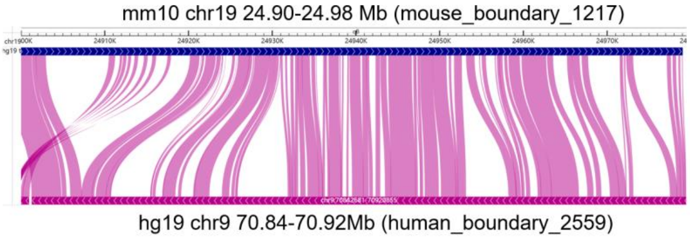

The human_boundary_2559 and human_boundary_1311 are clustered together, and the genome alignment confirms the result. Both TAD boundaries were aligned with a similar region on mouse chromosome 19, spanning approximately 24.90 to 24.98 Mb.

The effect of different aligners on the Tcbf results

For the whole genome alignment, different aligners will report variable results. We found the 126 TAD boundaries produced by liftOver were missing in the Tcbf due to the difference of used aligner. In this analysis, we aligned the human genome sequence of 126 TAD boundaries to the mouse mm10 genome with Tcbf default aligner minimap2 (parameters with asm20), blat (recommended human-mouse comparison parameters with -tileSize=11 -stepSize=11 -oneOff=0 -minMatch=2 -minScore=30 -minIdentity=90 -maxGap=2 -maxIntron=75000) and lastz (E=30 H=3000 K=5000 L=5000 M=10 O=400 T=1). The results show that each aligner has its own advantages and limitations.

a. In the minimap2 result, we only found 17 TAD boundaries had alignments with mm10. The average alignment length in each TAD was only 640bp and the maximum alignment length was 1900bp. The default alignment filter length of Tcbf is set to 2000bp to ensure the discovery of the real result for conserved TAD boundaries. In addition, we checked the 139 conserved TAD boundaries that were discovered by

Tcbf sequence alignment approach and liftOver, and found the average alignment length was 10451bp. So the Tcbf algorithm considered the 126 TAD boundaries were not conserved with human and mouse. In fact, the Tcbf provides two optional parameters to adjust the alignment parameter and filter the alignment length. We advise testing custom parameters to fine-tune the analyses. The default settings can be used to generate results quickly, but may not be best suited for each case-by-case analysis.

b. In the blat result, a total of 126 TAD boundaries have alignments in mm10. blat is useful for capable of mapping sequences with abundant gaps or spliced sequences from discontinuous genomic regions, but the result contains highly fragmentary alignment. The average alignment length is only 65bp and the maximum single alignment length is only 2288bp. However, blat cannot meet the speed requirements needed for large-scale analysis and regularly updated annotations. The running time is over 35 hours for aligning the 126 TAD boundaries (only 4.5% of human TAD boundaries) to the mm10 genome. Although users can split files to leverage multiple processes for acceleration, this is also unacceptable computing time for large-scale pan-3D genome research.

c. In the lastz result, we found 121 TAD boundaries have alignments with mm10. The average single alignment length is 3346bp, and the maximum alignment length is 14849bp. In addition, the runtime is about 40 minutes. The lastz aligner appears to perform well in terms of alignment length and offers a more efficient runtime compared to blat, so we add the optional aligner lastz for higher accurate comparison to small-scale pan-3D genome research. Users can custom aligner parameters to get much better results with longer runtime. Despite the better alignment length and runtime, it is essential to consider that the lastz aligner utilizes specific parameters. These settings are optimized for the particular comparison between human and mouse genomes. For other genome comparisons or specific research requirements, it may be necessary to fine-tune the parameters further.
