# Supplementary material for: Tcbf: a novel user-friendly tool for pan-3D genome analysis of topologically associating domain in eukaryotic organisms
Source: Bioinformatics. 2023 Sep 19;39(9):btad576. doi: 10.1093/bioinformatics/btad576 (PMC10539074; doi:10.1093/bioinformatics/btad576)
Supplement: btad576_Supplementary_Data [file btad576_supplementary_data.zip › Supplementary_File_1.pdf]

Tcbf User Manual

Topologically associating domain Conservative Boundary Finder (Tcbf) is a Python/R pipeline, which is used to analyze the conservation of topologically associating domain (TAD) boundaries between multiple species, which can serves the field of pan-3D genomic. TAD is the three-dimensional genomic characteristic structure of eukaryotic organisms. Its boundaries are conserved between different species and tissues. Conservative or dynamic topologically association domain is related to biological processes such as species evolution, reproductive development, and immune response. Tcbf can evaluate the degree of conservation of three-dimensional spatial structure among multiple species, and check its corresponding relationship in different species, which helps users understand the relationship between three-dimensional genome characteristic structure and basic life activities in a deeper way, and also supports the visual display of the results of the conserved topologically associating domain.

SOURCE CODE: https://github.com/TcbfGroup/Tcbf.

CONTACT: hexin9808@gmail.com or mjwang@mail.hzau.edu.cn

1. Introduction

TADs are fundamental regulatory chromatin structures and are largely conserved across tissues and species. We developed a Python/R pipeline Tcbf to identify the conservative TAD boundary between multiple genomes.

2. Usage

2.1 Installation

2.1.1 Python and R install

Before running Tcbf, users need to install Python and R language on the Linux operating system. For details, please refer to Python (https://www.python.org) and R (https://cran.r-project.org/) official sites.

2.1.2 R language and the following packages

```
install.packages(BiocManager)
install.packages(GenomicRanges)
install.packages(plyranges)
```

2.1.3 Install Tcbf from source

```
git clone https://github.com/TcbfGroup/Tcbf
cd Tcbf
pip install -r requiresments.txt
python setup.py install
```

2.2 Prepare input file

The configuration file named config.txt is a table format file with four columns as follows:

```
Genome1.fasta	genome1_tad.txt	genome1	genome1.gff3
Genome2.fasta	genome2_tad.txt	genome2	genome2.gff3
Genome3.fasta	genome3_tad.txt	genome3	genome3.gff3
```

The first column is the path to the reference genome file stored in FASTA format. For species that has a complete genome chromosomal assembly, it is recommended to delete all scaffolds.

The second column is the location of the TAD annotation file path.

The third column is the number or name of the species or material.

The fourth column is the gene annotation file in gff3 format for the species.

We use the result from HiTAD as the TAD annotate file.

```
Chr01 0         230000
Chr01 230000    395000
Chr01 395000    460000
```

2.3 Usage example

You can test the Tcbf pipeline with the example.

```
bash example/download_exampleData.sh
tcbf run -c example/config.txt -o test
```

-c the config.txt file   [required]

-o --output output path. [required]

-d –distance TAD boundraies range. [deafult:40000]

-p threads number. [default:0.]

--maxgap [deafult:40000]

-minimaplength [deafult:2000]

Advanced

1. For different species comparisons, we provide different minimap2 comparison parameters, which depend on the MASH distance of the two species.

**MASH**	**Parameters**	**Species 1**	**Species 2**
**<0.001**	**asm5**	**hg38**	**hg37**
**0.001~0.1**	**asm10**	**hg38**	***P. troglodytes***
**0.1~0.2**	**asm20**	**hg38**	***M. musculus***
**>0.2**	**sr**	**hg38**	***G. gallus***

2. For small-scale cross-species comparisons, we provide precise lastz aligner, the preset parameters is “E=30 H=3000 K=5000 L=5000 M=10 O=400 T=1”, but we strongly advise to test custom parameters to finely tune the analyses. These presets are meant to be used to generate results quickly, and might not be best suited for your purpose.

3. For HPC users who have multiple computation nodes, we provide a mode for split work to accelerate work.

```
tcbf run -c example/config.txt -o test –only_print_command
```

The Tcbf will print the command on the screen and needs to be submitted by users.

```
### Step1
tcbf extract-boundary -t data/human.txt -g data/human.Chr.fa -d 40000 -p human -o test
tcbf extract-boundary -t data/mouse.txt -g data/mouse.Chr.fa -d 40000 -p mouse -o test
tcbf extract-boundary -t data/macaque.txt -g data/macaque.Chr.fa -d 100000 -p macaque -o test
### Step2
tcbf process-alignment -q human -t mouse -o test --threads 0 -aligner minimap2 -minioverlap 100
tcbf process-alignment -q human -t macaque -o test --threads 0 -aligner minimap2 -minioverlap 100
tcbf process-alignment -q mouse -t human -o test --threads 0 -aligner minimap2 -minioverlap 100
```

```
tcbf process-alignment -q mouse -t macaque -o test --threads 0 -aligner
minimap2 -minioverlap 100
tcbf process-alignment -q macaque -t human -o test --threads 0 -aligner
minimap2 -minioverlap 100
tcbf process-alignment -q macaque -t mouse -o test --threads 0 -aligner
minimap2 -minioverlap 100
### Step3
tcbf third-construct-tad-network -o test
```

2.4 Output format

For conserved TAD boundaries among species, we provide a table file to show the clustering results. This table has one TAD boundary group per line and one species per column and is ordered from the largest orthogroup to the smallest.

	Human	Macaque	Mouse
Group1	Human_boundary_1386	Macaque_boundary_1415	Mouse_boundary_43
Group2	Human_boundary_480	Macaque_boundary_846	Mouse_boundary_2384

2.5 Visualization

The user can input a region of the genome, and the software can automatically obtain the corresponding 3D structural relationship. The red part in the figure represents the TAD range, and the yellow rectangle represents the TAD boundary. Light blue indicates collinear pairs.

```
tcbf    plot-syn-pair -o out –reference human —-chrom chr7 –start 127910000
–end 131410000 –plot test.pdf
```

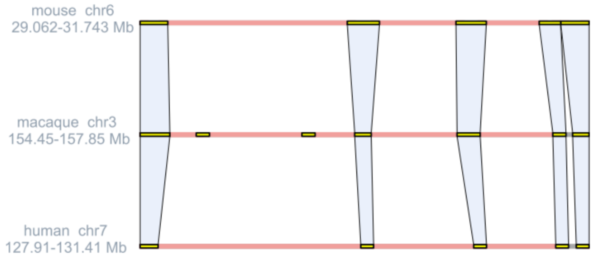

For heatmap visualization of multiple species, please refer to the example script. (https://github.com/TcbfGroup/Tcbf /blob/main/example/heatmap.R)

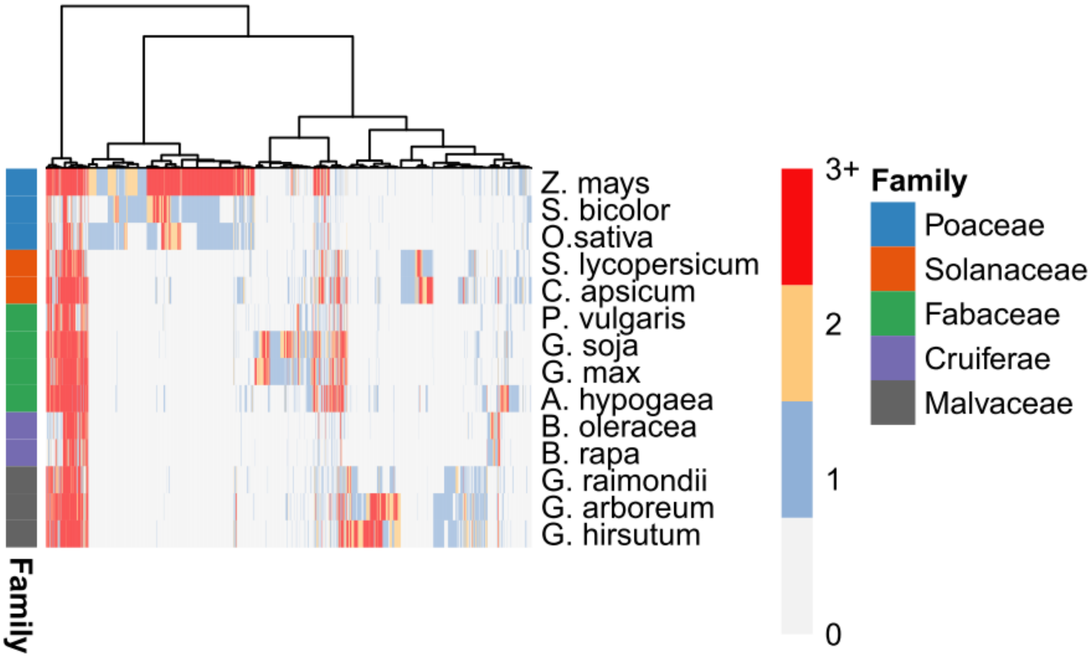

The good performance of Tcbf depends on a few conditions. (1) It is challenging to accurately detect the conserved TAD boundaries in highly fragmented genomes with low-quality gene annotations. To minimize bias, we recommend using genomes with removal of unplaced contigs and scaffolds and high gene annotation quality. With the rapid advances in sequencing technologies for the assembly of high-quality reference genomes, this should not be an issue for most species. (2) In the TAD boundary identification process, we recommend using high-quality Hi-C or similar data with the same resolution in different species to reduce the input data bias of TAD coordinates. (3) For input data with variable genome sizes, users may select an appropriate TAD boundary range through the average TAD size to improve the quality of the results.

3. License

MIT License

Copyright (c) 2023 Huazhong Agricultural University

Permission is hereby granted, free of charge, to any person obtaining a copy of this software and associated documentation files (the "Software"), to deal in the Software without restriction, including without limitation the rights to use, copy, modify, merge, publish, distribute, sublicense, and/or sell copies of the Software, and to permit persons to whom the Software is furnished to do so, subject to the following conditions:

The above copyright notice and this permission notice shall be included in all copies or substantial portions of the Software.

THE SOFTWARE IS PROVIDED "AS IS", WITHOUT WARRANTY OF ANY KIND, EXPRESS OR IMPLIED, INCLUDING BUT NOT LIMITED TO THE WARRANTIES OF MERCHANTABILITY, FITNESS FOR A PARTICULAR PURPOSE AND NONINFRINGEMENT. IN NO EVENT SHALL THE AUTHORS OR COPYRIGHT HOLDERS BE LIABLE FOR ANY CLAIM, DAMAGES OR OTHER LIABILITY, WHETHER IN AN ACTION OF CONTRACT, TORT OR OTHERWISE, ARISING FROM, OUT OF OR IN CONNECTION WITH THE SOFTWARE OR THE USE OR OTHER DEALINGS IN THE SOFTWARE
